# The SPIRIT Checklist—lessons from the experience of SPIRIT protocol editors

**DOI:** 10.1186/s13063-022-06316-7

**Published:** 2022-04-27

**Authors:** Riaz Qureshi, Alexander Gough, Kirsty Loudon

**Affiliations:** 1 Department of Ophthalmology, School of Medicine, University of Colorado Anschutz Medical Campus, Aurora, USA; 2 Institute of Applied Health Research, University of Birmingham, Birmingham, UK; 3 Independent researcher, Edinburgh, UK

**Keywords:** Randomised controlled trials, SPIRIT, Protocol, Structured template, Peer reviewers, Trialists, Editors

## Abstract

Crystal clear RCT protocols are of paramount importance. The reader needs to easily understand the trial methodology and know what is pre-planned. They need to know there are procedures in place if there are, for instance, protocol breaches and protocol amendments are required, there is loss to follow-up and missing data, and how solicited and spontaneous reported adverse events are dealt with. This plan is important for the trial and for the results that will be published when the data is analysed. After all, individuals have consented to participate in these trials, and their time and their well-being matter. The Standard Protocol Items: Recommendations for Interventional Trials (SPIRIT) provides guidance to structure RCT protocols and ensures all essential information is included. But sadly, not all trialists follow the guidance, and sometimes, the information is misunderstood. Using experience peer-reviewing for *Trials* over the last 2 years, we have prepared information to assist authors, peer reviewers, editors, and other current and future SPIRIT protocol editors to use the SPIRIT guidance and understand its importance.

## Introduction

The Standard Protocol Items: Recommendations for Interventional Trials (SPIRIT) statement was published in 2013 as “evidence-based recommendations” for the minimum information that should be provided to describe a randomised controlled trial (RCT) protocol [1]. *Trials*, like many journals, endorsed and adopted the checklist, requiring that unstructured protocols published in *Trials* must be accompanied by a complete SPIRIT checklist.

In September 2019, due to inconsistency in the standard of protocols submitted and the large number of submissions, *Trials* piloted the use of dedicated SPIRIT protocol editors to review the submissions that claimed to already have undergone peer review as part of their funding application with a specific focus on the clarity and comprehensiveness of SPIRIT reporting. As a result of the pilot’s success, the project was expanded, and there are now 18 SPIRIT protocol editors working to improve the standard of protocols published in *Trials*. Often, reviews by these editors note missing information that has not been picked up during routine peer review.

In November of 2019, an alternative submission type was introduced which follows a structured template that includes all SPIRIT items and does not require an associated checklist. The use of the *Trials*-structured protocol can improve the flow of protocols and ensure that all information is included, as well as enabling readers to easily search for specific items in a protocol [2]. This framework is particularly useful to readers for items which can be lost in the middle of some protocols which have few headings or are written narratively, such as item 8 (specific trial design) and item 14 (sample size).

The SPIRIT Checklist has now been translated into Chinese, French, Italian, Japanese, Korean, and Spanish, so although *Trials* is an English language publication, authors have an opportunity to read an accepted translation and understand exactly what each of the SPIRIT items entails [3]. Additionally, many extensions have been developed for SPIRIT to accommodate the differences in requirements for various subspecialties of medicine and subtypes of trials (Table 1).

**Table 1:** Extensions to the SPIRIT Checklist

• SPENT 2019 – SPIRIT extension and elaboration for n-of-1 trials [4]	
• SPIRIT-PRO – extension explanation and elaboration: guidelines for inclusion of patient-reported outcomes in protocols of clinical trials [5]	
• SPIRIT-AI – guidelines for clinical trial protocols for interventions involving artificial intelligence [6]	
• SPIRIT-TCM – Standard Protocol Items for Clinical Trials with Traditional Chinese Medicine 2018: recommendations, explanation, and elaboration [7]	
• SPIRIT-Path – guidelines for cellular and molecular pathology content in clinical trial protocols [8]	
• SPIRIT-ROUTINE – a study protocol for the development of a SPIRIT extension for trials conducted using cohorts and routinely collected data (under development) [9]	

Although the checklist was published in 2013, as with many reporting checklists such as Preferred Reporting Items for Systematic Reviews and Meta-Analyses (PRISMA) and Consolidated Standards of Reporting Trials (CONSORT), the adherence and compliance among publications have seen only moderate improvement. A recent methodological study compared the overall proportion of checklist items adequately reported in RCT protocols published before and after the SPIRIT statement, respectively in 2012 and 2019 [10]. The investigators found an average of 57% of items were adequately reported in 2019 protocols, as compared with 48% in protocols from 2012 [10]. While this is a mean improvement of 9%, for the 55 items in the SPIRIT checklist, the results suggest that after 6 years, not even two-thirds of all items are adequately reported, and the investigators found no protocols among the 150 from 2019 addressed all items [10]. Understanding the features of protocols associated with non-adherence and how reporting standards may be improved is still an area of active research interest [11].

With regard to the SPIRIT reporting experience at *Trials* specifically, an editorial was published in 2017 [12] that addressed the questions the following: “What is expected in a protocol submission?”, “When to submit a protocol for publication”, “What is the purpose of peer review of protocol submissions to *Trials* journal?”, and “Can we improve the process?” The information and advice in the 2017 editorial are useful supplements to the original information in the original SPIRIT statement and its associated explanation and elaboration documents [1, 12, 13]. The editorial made four suggestions to improve the peer review process of protocol submissions to *Trials*: (1) that protocol authors optimise the quality of their reporting and adhere to journal guidelines for submission, (2) that editors and peer reviewers of the journal familiarise themselves with all the journal guidelines, (3) that more contributions be made from the trials community as editors and reviewers, and (4) that peer reviewers continue to provide constructive comments to improve the quality of reporting [12].

We believe that the various SPIRIT guidance documents and editorials, as well as the extensions, should be considered complementary and required reading for any protocol authors, regardless of whether the protocol is submitted for publication or not. The SPIRIT explanation and elaboration documents provide detailed descriptions, rationale, and examples for all items that are important in describing the design and conduct of a trial, in general. The extensions provide additional insight and recommendations about the items that are unique to certain trial designs and are not covered by the primary SPIRIT documentation. The editorial by Li et al. provides insight into the need for transparency and accountability in reporting trial design and suggests a path towards reaching these goals. Lastly, this current article provides additional guidance on some SPIRIT items that are commonly misinterpreted or missed entirely to hopefully improve trialists’ and editors’ understanding of how to make sure a protocol does not inappropriately ignore relevant aspects of the trial’s design and conduct.

We (i.e. the authors of this editorial) are designated protocol editors with the journal and have between ourselves submitted over 1876 [466 RQ + 356 AG + 1054 reviews KL] reviews for 1110 [240 RQ +216 AG + 654 KL] unique trial protocols submitted to *Trials* since fall of 2019. Each of us has received extensive training in trial design and analysis methods through our various degrees and work experiences, including with the Johns Hopkins Center for Clinical Trials and Evidence Synthesis, Birmingham University Medical and Dental School, Cambridge University Department of Veterinary Medicine, the Cochrane Collaboration, the University of Dundee and Tayside Clinical Trials Unit, and the Edinburgh Clinical Trials Unit. Additionally, we have all received training and mentoring from senior *Trials* editors regarding the rationale and implementation of the SPIRIT Checklist, and we all have had Good Clinical Practice training. We believe our training and number of reviews completed give us a unique perspective on common issues and opportunities for improvement in the reporting of trial protocols.

The aims of this article are to describe common errors in the submission of protocols and to make suggestions to improve the quality of the submitted protocols, informed by our experience of reviewing submissions to *Trials*. This information should be useful to authors, peer reviewers, editors, and other current and future SPIRIT protocol editors.

## What are the most common errors in SPIRIT Checklists?

In order to determine which SPIRIT items require special attention, we independently listed the 12 items which we each believed to be the most commonly inappropriately or inadequately addressed and requiring a comment. From this informal poll, we took any overlap in our listed items as being those requiring special clarification for protocol authors. The SPIRIT Explanation and Elaboration document contains a detailed explanation of why each of these items is necessary and how the information is useful [1]. Rather than repeating what is already written and recommended as reading for authors, we present some of our own insights into these commonly inadequately addressed items as to why they are often unaddressed and how authors may consider them.

Item 5d—Composition, roles, and responsibilities of the coordinating centre, steering committee, endpoint adjudication committee, data management team, and other individuals or groups overseeing the trial, if applicable.

While not every trial has a need for multiple groups involved in trial oversight, such as a data monitoring committee, endpoint adjudication committee, or even an official steering committee, there needs to be someone, or some group, tasked with managing the trial. This item is often left incomplete or as “not applicable” because authors assume it does not need explanation if they do not have any formal committees. In fact, if there are no such formal groups involved in trial oversight, it is just as important for the protocol to describe who is in charge of the trial and making all relevant decisions, in what capacity they are acting as well as their roles and responsibilities, and why it was deemed not necessary to create any of the aforementioned formal committees. It may be that the trial investigators are handling all aspects of the trial management, from monitoring enrolment and training of study staff to checking the data quality, but if this is the case, it needs to be clearly stated and the lack of other groups rationalised.

Item 8—Description of trial design including type of trial (e.g., parallel group, crossover, factorial, single group), allocation ratio, and framework (e.g., superiority, equivalence, noninferiority, exploratory).

The most common omission in this item is the failure to specify the framework of the trial. While most randomised controlled trials have a superiority framework (i.e. they aim to prove that one treatment is superior to another or to a placebo), some trials aim to prove a non-inferiority (that a new treatment is not unacceptably worse than an existing treatment) or equivalence (whether a new treatment is equivalent to an existing treatment) framework. The choice of the framework has important implications for many aspects of the trial’s design including the hypotheses, the expected effect sizes, the sample size, the analytical considerations such as handling of missing data, and the interpretation of the statistical results. A detailed discussion is beyond the scope of this article, but further information can be found in numerous published articles such as Stefanos et al. [14]

Item 12—Primary, secondary, and other outcomes, including the specific measurement variable (e.g., systolic blood pressure), analysis metric (e.g., change from baseline, final value, time to event), method of aggregation (e.g., median proportion), and time point for each outcome. Explanation of the clinical relevance of chosen efficacy and harm outcomes is strongly recommended.

Common omissions in this item include the analysis metric (e.g. comparison at a specific time point, comparison of the change from baseline) and the method of aggregation (e.g. comparison of the mean/median or the proportion who experience a dichotomized outcome). Complete specification of the planned outcomes is important because not addressing these items can lead to ambiguity in the interpretation of the expected outcome. However, another common and important error concerning outcome specification is the nomination of multiple primary outcomes without accounting for this in the statistical plan. This multiplicity greatly increases the probability that a significant result is due to random chance. Multiple primary outcomes can appear in a protocol both because different measurement variables are nominated and the same measurement variable is nominated at multiple time points. While it is common that multiple primary outcomes are not adjusted for, even in published research [15], trial investigators should strongly consider whether they have multiple primary outcomes of equal importance and adjust their analyses accordingly or if there is a single designated primary outcome followed by multiple secondary outcomes.

Item 14—Estimated number of participants needed to achieve study objectives and how it was determined, including clinical and statistical assumptions supporting any sample size calculations.

While most trial protocols do include a section to state their sample size and some of the assumptions to accompany the final number (e.g. power and alpha), many protocols fail to include all necessary elements for estimating the sample or provide the rationales and sources to support the assumptions regarding detectable effect size. It is not enough to state the given sample size for the trial. The authors must state the software and hypothesis test used to generate the sample size and all parameters used in the generation of the sample size, provide sources—or rationale if no sources exist—for estimates of effect, any additional assumptions for non-two-arm parallel designs (e.g. intracluster correlation coefficient for cluster trials, clinically relevant non-inferiority margins for non-inferiority trials, etc.), clearly specify which outcome is being used to inform the estimation (and justification if it is not the primary outcome), and note whether the final estimate includes any accounting for potential loss-to-follow-up. Protocol authors often fail to include all of these necessary components. A good rule of thumb for protocol authors to follow is to ensure that the estimate can be reproduced (or at least approximated) with what is given in the protocol. Additionally, if a trial protocol does not have a formal sample size estimation (e.g. some phase II trials), it is still important that the authors provide their reasoning and support for the target sample size.

Item 20c—Definition of analysis population relating to protocol non-adherence (e.g., as randomised analysis), and any statistical methods to handle missing data (e.g., multiple imputation).

Many protocols include the name for their analysis population(s), such as intention-to-treat; however, it is very common that protocols fail to define which participants exactly are included in all analysis populations. It is important to define the analysis populations in the context of the trial because the name itself may be incorrectly used. For example, oftentimes, an analysis is called “ITT” when it is actually a modified ITT, that is, it applies some additional criteria for inclusion on top of being randomised such that it is no longer a pure as-randomised analysis (e.g. “We included all participants who attended at least three out of four follow up visits in the groups to which they were assigned”). Specificity helps the readers know what exactly was planned for the trial. Additionally, protocols must specify the planned methods for assessing any missing data during the analyses.

Item 21b—Description of any interim analyses and stopping guidelines, including who will have access to these interim results and make the final decision to terminate the trial.

Interim analyses allow the early termination of trials because of unacceptable harms (i.e. adverse events), evidence of futility, or even overwhelming evidence of efficacy meaning it would be unethical to deny the control arm the effective treatment. However, unplanned interim analyses risk damaging the trial integrity, for example, by breaking the blinding, and may also risk an unjustified rejection of the null hypothesis (i.e. a type I error). Stopping guidelines must be carefully formulated to take into account the risk of taking a decision to stop the trial based on incomplete data. Further information on the interim analysis and stopping guidelines can be found in Kumar and Chakroborty [16].

Item 22—Plans for collection, assess, reporting, and managing solicited and spontaneously reported adverse events and other unintended effects of trial interventions or trial conduct.

Many protocol authors will give a definition of harms to describe what they might consider to be adverse events (AEs) or serious adverse events (SAEs) and include a note about reporting harms to Institutional Review Boards or Data Monitoring Committees (DMCs). However, the description of harm assessment in protocols is often incomplete. If there are any potentially expected harms given prior experiences or knowledge of the intervention(s) being assessed, these should be listed. The authors should also note if unexpected harms will be collected and define how all harms will be collected: systematically (i.e. solicited from all participants in a standardised manner) or non-systematically (e.g. unsolicited collection using participant’s spontaneous report). It is also good for investigators to note whether harms will be classified or codified according to any standard language (e.g. Medical Dictionary for Regulatory Activities (MedDRA) or Common Terminology Criteria for Adverse Events (CTCAE)), as well as the plans for reporting harms in trial publications (e.g. whether all collected harms will be reported or only a subset that meets specific criteria). All of these details about harms are often missing from trial protocols, but they are important for readers who want to understand how a trial assessed harms. Special consideration of these details should be given to trials that claim to assess the “benefit and safety” of an intervention.

Item 25—Plans for communicating important protocol modifications (e.g., changes to eligibility criteria, outcomes, analyses) to relevant parties (e.g., investigators, REC/IRBs, trial participants, trial registries, journals, regulators).

Many protocols initially include this item as “not applicable” under the assumption that no modifications are planned. However, the item is always applicable as it entails the plan for any possible changes that may be necessary over the course of the trial. After specification, many authors note that important protocol modifications will be notified to the ethics committee or trial registries, but it is also necessary to communicate the changes to all investigators (especially in multi-centre studies or trials with large numbers of investigators) and to participants if this impacts the treatment recommendations they should be following or may alter their appreciation of trial risks or other aspects which could lead to requiring the investigators to obtain an updated informed consent.

Item 26b—Additional consent provisions for collection and use of participant data and biological specimens in ancillary studies, if applicable.

In 2001, the Redfearn report into the Alder Hey organ retention scandal was published, in which the unauthorised removal and retention of human tissue and organs, including children’s hearts, were revealed (https://www.gov.uk/government/publications/the-royal-liverpool-childrens-inquiry-report). It is important for ethical reasons that investigators are open about their plans for the retention and future use of biological specimens and obtain consent for any plans for these tissues and organs. Also, many authors read item 26b as only being applicable to the additional use of collected biological specimens, and if there are none collected in the trial, they will leave this SPIRIT item as “not applicable”. There are two aspects to consider for this item, however. The first is that it also applies to participant data in general, that is, any data collected over the trial that might be used in later studies. The second is that if the item is not applicable, a statement should clearly state why (i.e. that no additional studies are planned and consent will not be obtained for that potentiality).

Item 30—Provisions, if any, for ancillary and post-trial care, and for compensation to those who suffer harm from trial participation.

Randomised trials have some risks to participants. As it is unknown whether one treatment is superior to another, some participants may receive an inferior treatment, or they may experience unexpected harms. It is important that clinical trials minimise participant harm where risk is possible, by providing ancillary and post-trial care, and if there is a significant risk to the participant, compensation may be appropriate.

Item 31c—Plans, if any, for granting public access to the full protocol, participant-level dataset, and statistical code.

This item is often left as not applicable because protocol authors may believe that the item is specific to the protocol itself and because there is no data associated with the protocol, the item is therefore not applicable. However, this item is always relevant and applicable for a trial protocol as this is the declaration of whether the trial data, once completed, will be shared or made available to the public. This item should always have a statement at least describing whether trial data will be shared and how it can be accessed. In 2018, the International Committee of Medical Journal Editors (ICMJE) stated that manuscripts submitted must contain a data sharing statement and promoted sharing of de-identified data [17]. Note that it is acceptable for investigators to not share the data in some way, although it is greatly encouraged and may be required depending on the source of funding. Even if the data will not be shared, this SPIRIT item is still applicable and should be addressed with a statement that no trial data will be made available.

These SPIRIT items are the most commonly misunderstood by protocol authors according to our subjective assessment; however, there are many other common comments that are raised to address issues with SPIRIT reporting in submitted protocols. Table 2 contains a list of common comments for protocols that can be used by editors and peer reviewers at *Trials* if a protocol fails to adequately address the SPIRIT guidelines. Authors of protocols wishing to submit to *Trials* should take careful note to address these items.

**Table 2:** Common comments that are made on many initial submissions

SPIRIT item	Common comment
2b	“The trial is registered in [*trial registry other than ICTRP*]. Please either include a supplemental table with all items from the WHO Trial Registration Data Set, or else a statement affirming whether all items can be found in the protocol. Alternatively, simply state: ‘Please refer to Item 2a and registration in the EU Clinical Trials Register https://www.clinicaltrialsregister.eu/’ (or whichever is applicable) and provide a link to your trial.”
5c	“Please note the sponsor/funder’s role in any of the design, conduct, analysis, or future writing and publication. An example of what has been written is: ‘The sponsor played no part in study design; collection, management, analysis, and interpretation of data; writing of the report; and the decision to submit the report for publication.’”
5d	“Regarding trial oversight, aside from [*any already listed groups*], are there any study teams (e.g., Steering Committee, Data Monitoring Committee, Data Quality Committee, Stakeholder and Public Involvement Group, etc.) involved in monitoring the progress of the trial (e.g., checking recruitment, training staff, periodic auditing of data quality, interim analyses, etc.), and if so, what is the composition of these groups and what are their roles and responsibilities? There will always be a core group running the trial day-to-day and providing organisational support for the trial. This SPIRIT item does not require names, but information such as how often they will meet is useful. If there are no such groups aside from the core group, please provide a note for why they were deemed not necessary.
6b	“Please provide more detail and rationale for your choice of comparator. Note that all randomised controlled trials have a comparator, even if it is placebo or standard care.”
8	“Please be more specific and complete in the description of the trial design framework (e.g. superiority, equivalence, non-inferiority, exploratory). For example see Dunn Trials 2018;19(1):499” [18].
11b	“Are there any reasons why participants might discontinue or modify the intervention? For instance, please state criteria for discontinuing or modifying allocated interventions for a given trial participant (e.g drug dose change in response to harms, participant request, or improving/worsening condition). The protocol may state ‘There will be no special criteria for discontinuing or modifying allocated interventions.’”
11d	“Aside from the trial interventions, are there any other treatments or therapies that participants are allowed or not permitted to use? The protocol could state that implementing X or Y will not require alteration to usual care pathways (including use of any medication) and these will continue for both trial arms.”
12	“Please fully define all your outcomes following the framework described in Zarin NEJM 2011;364:852-60 and Saldanha PlosOne 2014;9(10):e109400. Your outcome definition should include these 5 elements: the domain (name of the outcome), specific measurement, metric (i.e., will the difference at a point in time between groups be assessed, or the difference in the change in score between two groups, etc.), method of aggregation (e.g., mean, median, proportion, etc.), and time point (i.e., which of the times at which the outcome will be assessed is of interest for the comparison). For example, [*missing details for at least one trial outcome that is incompletely or ambiguously described*]” [19, 20]
13	“Please include a SPIRIT Figure with the protocol to present the timing of all assessments and measures in the trial. The study flow diagram currently included is helpful, but not sufficient. Please see the SPIRIT explanation and elaboration guidance (Chan, BMJ, 2013; 346: e7586) and other protocols published in *Trials* for examples.”
14	“The sample size estimation requires elaboration. What estimates and assumptions were used in generating this sample size? What is the minimal difference you will be able to detect and with what power and at what level of significance? Which outcome was used to inform the sample size and if it is continuous, what are the expected mean(sd) for each group being compared, with references/rationale to support the estimation?”
15	“Please insert into the protocol information on recruitment strategy to ensure adequate participant enrolment to reach sample size. The protocol must state how you will recruit and the anticipated recruitment period. Additionally, are there any extra processes or measures in place to improve recruitment and ensure an adequate enrolment is met?”
16a-c	“The randomization and allocation processes could be elaborated. What program is used to generate the random sequence and who generates it? Is the randomization simple or is there any blocking or stratification (or other adaptation) used? Is the allocation sequence concealed before randomization and how do the people performing the allocation obtain each subsequent assignment? Who performs the allocation?”
17a	“The trial is described as double-blind, which is ambiguous, and more than two parties are blinded. Consider revising the title and describe all the specific blinded parties early in the protocol.” “For the description of blinding, please also note whether statisticians and outcome assessors will be blinded to treatment, and if not, please provide a rationale.”
17b	“If the trial includes any blinding, N/A is not acceptable: please describe any circumstances under which involved parties (e.g., clinicians, coordinator, etc.) may be unblinded. Alternatively, the protocol could state ‘We do not anticipate any requirement for unblinding but if required, the Trial Manager, Data Coordinator, Clinicians will have access to group allocations and any unblinding will be reported.’ or, if the trial is not blinded: ‘The design is open label so unblinding will not occur.’”
18a	“The data collection and management processes could be elaborated. How will data be collected and are the forms pre-existing or created by the investigators? Are there any processes in place during data collection/entry to ensure data are complete and accurate?”
18b	“Given the anticipated drop-out as described in the sample size section, are there any plans or measures in place to improve participant retention and minimise loss to follow up?”
19	“Please give details for data entry, coding, security, and storage, including any related processes to promote data quality (e.g. double data entry, range checks for data values). Reference to where data management procedures can be found, if not in the protocol. For instance, will paper based and electronic data entry be used? Who will collect data? And who will enter the data into the database for screening and randomisation purposes? If paper forms are used, will you ensure that the paper-based Case Report Form (CRF) data are delivered securely to the Trial Office for data entry?”
20b	“If there are any secondary/subgroup/interim analyses planned for the trial, please include a description of these including rationale and the methods of analysis. If none are planned, please include a statement to that effect.”
	“For the analysis section, please define who is included in all trial analysis populations (e.g., ITT, modified-ITT, full analysis set, per protocol, safety) in the context of the trial (i.e., how will you analyse those that are randomised to the intervention but do not adhere to the intervention?). Additionally, please include a statement about how missing data will be handled in the trial.”
21b	“The description of interim analyses requires more detail. Please include stopping guidelines in relation to your interim analysis, as well as who will conduct the analysis and how the results will influence decisions to continue or terminate the trial.”
22	“Regarding harms, are there any harms that may be potentially expected given previous experiences with the study intervention(s)? If so, please list them.” “Will unexpected harms also be collected and how will all harms be assessed: systematically (i.e., using a standardised approach for all participants) or non-systematically (i.e., spontaneous reporting from participants or asking about non-specific events)?” “Will harms be coded (i.e., standardised according to structured language such as Medical Dictionary for Regulatory Activities (MedDRA) or Common Terminology Criteria for Adverse Events (CTCAE))?” “Additionally, what are the plans for reporting harms in trial publications (i.e., will you report all harms or only those which meet specific criteria)?”
25	“If there are any amendments required to the protocol over the course of the trial, how and to whom will these be communicated? For example, notifying the sponsor and funder first then the PI notifying the centres with a copy of the revised protocol sent to the PI to add to the Investigator Site File. You may also want to state that any deviations from the Protocol will be fully documented using a breach report form. You can also include that you will update the protocol in the clinical trial registry.”
26a	“Please give information on who was actually recruiting the participant and gaining informed consent. When and how is this done?”
26b	“If this item is Not Applicable because biological specimens will not be collected, it is important to state that ‘This trial does not involve collecting biological specimens for storage.’ Please also include a note about whether there are any plans for additional studies using the general participant data collected in this trial and whether consent will need to be sought to use that data.”
Item 27	“Please expand the description of how confidentiality will be ensured. Verify that data collected during the course of the research will be kept strictly confidential and only accessed by members of the trial team (or individuals from the Sponsor organisation or centre sites where relevant to the trial). Will participants be allocated an individual trial identification number and will participant’s details be stored on a secure database? Who will access rights to the data set? Will anonymised trial data be shared with other researchers to enable international prospective meta-analyses?”
Item 29	Statement of who will have access to the final trial dataset, and disclosure of contractual agreements that limit such access for investigators. Consider stating “Any data required to support the protocol can be supplied on request.”
30	“What are the provisions for post-trial care and compensation for any participants who suffer harm? The protocol could potentially state: ‘There is no anticipated harm and compensation for trial participation.’”
31b	“Please include a note about how authorship will be determined for future trial publications and any intended use of professional writers. Additionally, please ensure that the protocol states that ‘all authors read and approved the final manuscript.’”
31c	“Please include a statement about whether there are any plans to give public access to the data generated for this trial. If data will be made available (either at participant or summary level), please include a description of how the data can be obtained or requested. For example, ‘The datasets analysed during the current study are available from the corresponding author on reasonable request.’”
32	“Please include a copy of the informed consent documents provided to participants.”
33	For this SPIRIT item, please add information on plans for collection, laboratory evaluation, and storage of biological specimens for genetic or molecular analysis in the current trials and for future use in ancillary studies. OR state there will be no biological samples collected.

In addition to the subjective assessment of uncommon SPIRIT items, two authors (RQ and KL) have collected data from a set of protocols assessed during the piloting of the SPIRIT Reviewer/Protocol Editor program at *Trials*. This assessment, which was conducted 2 years ago with protocols submitted in 2019, reveals the same patterns and that many SPIRIT items remain problematic in that they are forgotten and left unspecified in initial submissions. Table 3 presents the items that were left unspecified or marked as “not applicable” with no explanation in more than 10% of a sample of 90 protocol submissions.

**Table 3:** SPIRIT Checklist items left unspecified in at least 10% of original protocol submissions (*n* = 90)

SPIRIT item	Number of submissions in which item was left unspecified	
	***n***	%
**Administrative information**		
2b – WHO registration data set	42	47%
3 – Protocol version and date	13	14%
5c – Role of sponsors	17	18%
5d – Trial oversight groups	32	36%
**Methods: participants, intervention, and outcomes**		
11b – Criteria for modification	25	28%
11c – Adherence monitoring	17	19%
11d – Concomitant care	25	28%
**Methods: assignment of interventions**		
17b – Unblinding procedures	50	56%
**Methods: data collection, management, and analysis**		
18b – Participant retention	16	18%
20b – Additional analyses	17	19%
20c – Analysis population and missing data	12	13%
**Methods: monitoring**		
21a – Data monitoring committee	22	24%
21b – Interim analyses	48	53%
22 – Harms assessment	13	14%
23 – Trial auditing procedures	42	47%
**Ethics and dissemination**		
25 – Protocol amendments	17	19%
26b – Consent for use in ancillary studies	73	81%
29 – Access to data	16	18%
30 – Ancillary and post-trial care	54	60%
31a – Dissemination of results	23	26%
31b – Authorship eligibility	45	50%
31c – Data sharing and access	48	53%
32 – Model consent form	46	51%
33 – Biological specimen procedures	77	86%

In addition to providing objective evidence that many SPIRIT items are inappropriately completed on the SPIRIT Checklist, our examination of 90 protocols also shed light on the reasons why items may be left unspecified as follows:

(i) Because they are truly not applicable to the trial (e.g. unblinding of participants and clinicians is not applicable if a trial is open-label)
(ii) Because they were not done in the trial (e.g. trial is low risk and investigators choose not to form a data safety and monitoring committee)
(iii) Because they were done but the authors did not include it in the protocol (e.g. authors will make data from the trial available upon request, but did not include such a statement in the protocol)

Most items that were left unspecified or marked as “not applicable” without rationale were actually applicable to the trials and revised protocols included missing descriptors. Of the protocols that left these items incomplete, only a few items were affirmed as actually being not applicable in at least 50% of protocols after revisions: 17b “unblinding procedures”, 21b “interim analyses and stopping guidelines”, 26b “additional consent for ancillary studies”, and 33 “procedures for handling biological specimens.”

## Non-SPIRIT issues

While reviewing submissions for compliance with the SPIRIT Checklist, problems with the submissions are often identified by the reviewers that are not directly SPIRIT related. One very common issue that necessitates significant revisions is the quality of the English language. We recognize the challenge in writing in a second language and respect those authors who submit to English publications when it is not their first language; however, clarity is important when describing specific aspects of trial design—for both readers and reviewers—and we highly recommend that professional translators and editing services be used. Another related issue is that of potential ethical issues in the design of trials which can be related to several SPIRIT items including the rationale for the design, choice of comparator, monitoring, and ethical approval. Most trials submitting a protocol for publication in *Trials* are already underway, restricting us from commenting on potential design problems; however, if a trial does not appear to have an ethical basis (e.g. an intervention is compared to a placebo without the current standard of care or instead of an existing proven treatment option, without explicit and clear rationale [21]), this can lead to requests for clarification and may even result in rejection. Lastly, *trial status* is often not correctly completed in protocols submitted to *Trials*, which can influence acceptability as *Trials* is committed to transparency and accountability in prospective trial design and does not accept protocols from trials which have completed recruitment. Authors should state the protocol version number and date, the date recruitment began, and the approximate date when recruitment will be completed.

## How can SPIRIT Checklist compliance be improved?

### Recommendations for authors

It is highly recommended that the explanation and elaboration document is read in conjunction with the SPIRIT Checklist [1]. This paper gives both the reason for the inclusion of the SPIRIT items as well as thorough clarification of what is required. The *Trials* editorial from Li et al. in 2017 also provides useful supplemental information [12].

We also highly recommended that the new SPIRIT template be used for submissions. The use of curly brackets allows the questions of the SPIRIT Checklist to be answered directly and clearly within the protocol manuscript, without the need to complete a separate checklist. All the guidance is “right there” for authors to read and save them looking it up. But the template must be strictly adhered to. Authors cannot remove SPIRIT items they believe to be not applicable, and they cannot combine items or change the order of the SPIRIT items. If they do, then the protocol will need to be edited and corrected for publication in this format.

### Recommendations for editors and peer reviewers

Editors and peer reviewers should be familiar with the SPIRIT guidelines and be aware of issues. Most trial protocols submitted to the journal should be sent to one of the 18 SPIRIT reviewers or protocol editors for review, since they often find issues with SPIRIT compliance that other peer reviewers have not commented on. Editors and peer reviewers should also be aware of non-SPIRIT issues that the SPIRIT protocol editors often pick up on, such as language, ethics, and problems with statistical analysis.

We recommend that editors and reviewers keep a copy of our Table 2 handy when reviewing protocols and use comments where appropriate, modifying any aspects as needed. We hope that this editorial serves as a useful reference to editors, peer reviewers, and authors alike. Tables 4, 5, and 6 include some of our personal recommendations that we encourage authors and editors to remember when writing and reviewing protocols.

**Table 4:** Riaz Pet Peeves

• SPIRIT items left blank or marked N/A without rationale	
• Page numbers missing from protocol but referenced in the SPIRIT Checklist	
• Failure to provide any details about the groups involved in trial oversight and management	
• Incomplete description of randomization and allocation processes	
• Not including enough detail or justification in the sample size calculation to allow replication	
• Not enough detail on how potential harms will be assessed	
• Failure to specify how authorship will be determined in future trial publications (including any use of professional writers)	
• Failure to include a model consent form or (alternatively) details about its contents	
• Failure to consider the collection of blood and biomarker levels as “biological specimens”

**Table 5:** Kirsty Pet Peeves

• Not reading guidance for SPIRIT items	
• If non-English speaker, not getting article copy-edited	
• If using the SPIRIT Checklist, putting in a page number but there is no information on this item at that page number or anywhere else in the protocol, not just slippage	
• Putting in “not applicable” in SPIRIT Checklist or template without an explanation for why N/A	
• Not clearly stating the sponsor of the trial	
• Not declaring the role of the funding body in the design of the study and collection, analysis, and interpretation of the data and in writing the manuscript	
• Not explaining the framework used for the trial (e.g. superiority, equivalence, noninferiority, exploratory)	
• Not offering to share anonymised trial data without prompting	
• Not stating that the protocol in the public clinical trial registry will be updated if amended	
• Not giving a date when it is anticipated the trial recruitment will finish	
• If using *Trials* structured template, changing the order of the SPIRIT items	
• If using *Trials* structured template, missing out SPIRIT items	
• If using *Trials* structured template, missing out headings	
• If using *Trials* structured template, duplicate information, so under more than one item and not edited—suggesting authors copy and pasting protocol that has already been written into the template	
• If using *Trials* structured template, refers to information under another item instead of inserting it in the correct item

**Table 6:** Alex Pet Peeves

• Items that are missing from the checklist or marked N/A without explanation	
• Identification of a location in the SPIRIT Checklist which does not provide information that answers the checklist question	
• Failing to identify whether the trial has a sponsor separate from the funder	
• Failing to provide a model consent form	
• Failing to describe the framework of the trial	
• Listing multiple primary outcomes without accounting for this in the statistical plan	
• Failing to say who is responsible for generating the allocation sequence, who is responsible for enrolling the participants, and who is responsible for obtaining informed consent	
• Failing to describe how important protocol modifications are communicated to interested parties	
• Failing to state whether there are any provisions for ancillary and post-trial care for any participants harmed during the trial	

Given the various existing documentation that has been published to improve the quality and comprehensiveness of SPIRIT reporting in trial protocols, it is surprising that compliance is not higher. Recent research on trial protocol reporting has shown a significant improvement in the overall proportion of protocol items that are addressed since the SPIRIT guidance was published; however, this increase was only by approximately 9%, and several items were found to be less commonly reported [10, 11]. Studies that have examined interventions to improve adherence to reporting guidelines in general have found many different types of interventions for different stages of the publication process [22–24]. Although the effectiveness of many has not been evaluated, and even fewer with RCTs, among those that have been tested, only a couple have shown promise including a completeness of reporting check by editors, as is currently done by *Trials* protocol editors [22, 25]. Additionally, qualitative studies into the reasons for author and editor adherence to reporting guidelines have revealed several factors that influence their use, and a similar assessment with specific relevance to trial protocols may provide targets for future interventions to further improve the quality and completeness of SPIRIT reporting [26].

## Conclusions

Publishing of trial protocols in advance of publishing results is necessary in order to make methods of investigation transparent, which is vital for the integrity of the scientific process. The SPIRIT Checklist was designed to improve the quality of reporting of protocols of randomised controlled trials, but despite detailed guidance being available, compliance with the requirements of the checklist remains poor. The advice in this article from experienced protocol editors should help authors and editors ensure that their manuscripts are compliant with the recommendations of the SPIRIT statement. This will enhance the transparency and completeness of published protocols, which will benefit not only the authors and editors, but also trial participants, sponsors and funders, ethics committees, peer reviewers, trial registries, journals, and other stakeholders.

## Data Availability

The datasets used and/or analysed during the current study are available from the corresponding author on reasonable request.

## Abbreviations

AEs: Adverse events
CTCAE: Common Terminology Criteria for Adverse Events
CONSORT: Consolidated Standards of Reporting Trials
DMC: Data Monitoring Committee
IRB: Institutional Review Board
ITT: Intention-to-treat
MedDRA: Medical Dictionary for Regulatory Activities
PRISMA: Preferred Reporting Items for Systematic Reviews and Meta-Analyses
REC: Research Ethics Committee
RCT: Randomised controlled trial
SAEs: Serious adverse events
SPIRIT: Standard Protocol Items: Recommendations for Interventional Trials
